# Clinical and radiological comparison between partial and complete resection of the anterior cruciate ligament in patients with mucoid degeneration of the anterior cruciate ligament: a controlled clinical trial

**DOI:** 10.1007/s00402-022-04741-6

**Published:** 2022-12-27

**Authors:** N. Oehler, M. Haenle, S. Vogt, F. Blanke

**Affiliations:** 1 Department of Knee-, Shoulder- and Hip-Surgery and Orthopedic Sports Medicine, Schön Klinik München-Harlaching, Munich, Germany; 2 Department of Orthopedic Surgery, University Rostock, Rostock, Germany; 3 Department of Orthopedic Sports Medicine and Arthroscopic Surgery, Hessing Stiftung Augsburg, Augsburg, Germany

**Keywords:** MDACL, Shrinking, ACL, Resection, Mucoid degeneration, Impingement

## Abstract

**Introduction:**

The pathology of a mucoid degeneration of the anterior cruciate ligament (MDACL) has been mentioned in several publications but due to its rare incidence it is not a well-known pathology. Partial or complete resection of the ACL is the option of choice after failed non-surgical treatment. However, the success rate of both surgical techniques and the subsequent risk of an ACL instability is not known. The purpose of this study was to compare the clinical and radiological outcome between partial resection and complete resection of the ACL in patients with MDACL.

**Materials and methods:**

Patients with MDACL verified by MRI and persistent knee pain were treated by partial (Group I) or complete resection (Group II) of the ACL and were included in a controlled clinical trial after unsuccessful conservative treatment for at least 6 months. Demographic, clinical and radiological data including the thickness of ACL, ACL/intercondylar ratio, patient’s age at the time of surgery, the presenting symptoms, range of motion and ligament stability assessed by the ACL ligament score (Lachman test) were collected. In addition, Tegner activity score and Lysholm score were evaluated preoperatively and at final follow-up after a minimum of 12 months.

**Results:**

At final follow-up with a mean of 16.8 ± 8.8 months (range 12–41; Group I: 18.3 ± 9.7 vs. Group II: 15.3 ± 8.0; ns), all patients were pain free. Postoperatively, positive Lachman tests were noted in all patients (100%) in Group II (*n* = 5 patients with grade II and *n* = 5 patients with grade III). In Group I, 8 patients (80%) showed a negative Lachman test (grade I) and 2 patients (20%) a slightly elongated Lachman test with a firm stop (grade II). The mean knee flexion at follow-up examination was 132° ± 7° (range 120°–140°; Group I: 129° ± 9° vs. Group II: 135° ± 4°; ns). In pairwise comparison, flexion angle increased significantly in both groups (Group I: *p* = 0.0124 and Group II: *p* < 0.001). Pairwise comparison of thickness of the ACL and ACL/intercondylar ratio prior to and post-surgery in Group I showed non-significant differences.

**Conclusion:**

Both arthroscopic debridement and complete resection of the ACL lead to improvement of clinical and radiological findings in isolated MDACL. However, complete resection of the ACL will result in higher instability. Therefore, partial resection might be the better treatment option, especially in young patients with MDACL.

## Introduction

The mucoid degeneration of the anterior cruciate ligament (MDACL) is a well-known pathology, first described in 1999 [1]. It means a severe thickening of the ACL, which leads to stress edema of the bone, restriction of movement due to an intercondylar impingement and posterior knee pain [2, 3]. The etiology is still controversial and involves degenerative and traumatic reasons as well as a synovial malformation [3]. MDACL is diagnosed by magnetic resonance imaging (MRI) and the prevalence ranges from 1.8 to 5.3% [4]. It is characterized by a celery stalk sign, and it is confirmed by tissue biopsy and histological examination [4, 5]. Treatment for MDACL usually starts non-surgically with anti-inflammatory drugs, steroid injections, or physiotherapy. In case of unsuccessful conservative treatment, the surgical interventions include arthroscopic debridement with partial resection of the ACL or complete resection of the ACL with or without delayed ACL reconstruction [3, 6]. The success rate of both surgical techniques and the subsequent risk of an ACL instability is not known. There are only a few case reports which report a satisfactory outcome with residual instability of partial or complete resection of the ACL in patients with MDACL [6].

Thus, the purpose of this study was to compare the clinical and radiological outcome between partial resection and complete resection of the ACL in patients with MDACL. Moreover, the extent of postoperative knee instability was assessed in all patients. We hypothesized that partial resection might be the treatment of choice with superior knee stability and similar reduction of intercondylar impingement and pain.

## Methods

### Study design and study group

A retrospective controlled cohort study was performed. All patients gave their informed consent and institutional review board approval was obtained. Inclusion criteria were patient age > 18 years, an isolated MDACL diagnosed by MRI or by histological evaluation and a subsequent surgical treatment by partial resection and radiofrequency ablation or complete resection of the ACL. Patients with radiologically apparent degenerative joint disease, cartilage lesions > grade II to ICRS or meniscal lesions were excluded from the study. Patients were treated initially with nonsteroidal anti-inflammatory drugs, steroid injections and physiotherapy for a minimum of 6 months before considering surgery.

### Surgical procedure

**Fig. 1:**
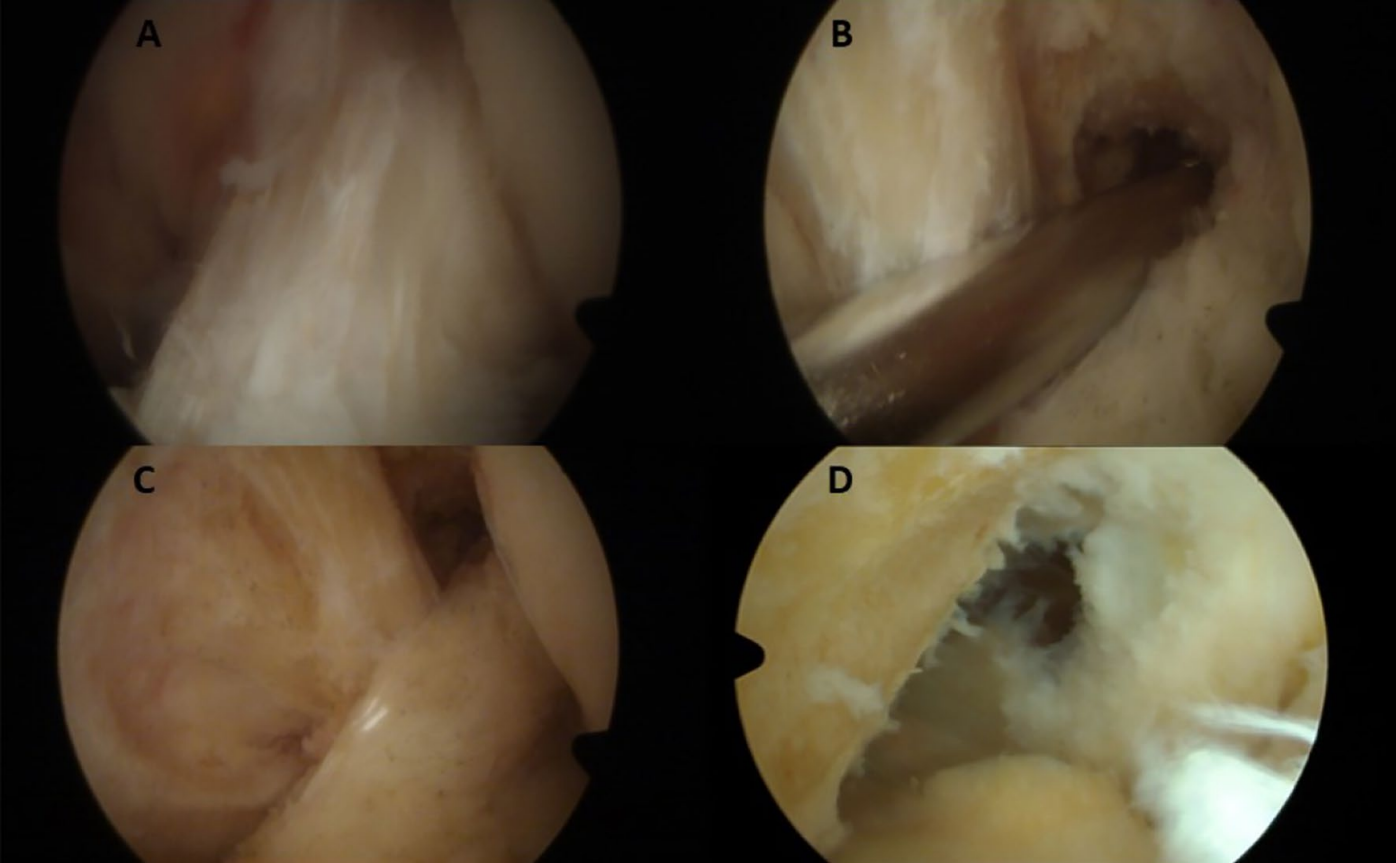
Surgical technique in MDACL. **A** Mucoid degeneration of ACL in the arthroscopic view. **B** Suction of the dorsal recessus. **C** Partial resection of ACL with ablation. **D** Resection of ACL

A single team of knee orthopedic surgery specialists (MH, SV, FB) performed all surgeries. During surgery, all compartments were explored to evaluate the state of menisci, ligaments and cartilage and mucoid degeneration of the ACL was confirmed (Fig. 1). Arthroscopic treatment of partial resection consisted of the removal of all mucoid degenerated tissue of the ACL and a ligament shrinking by radiofrequency ablation (Fig. 1). Arthroscopic treatment of complete resection was defined by removal of all parts of the ACL and debridement of the former footprints (Fig. 1). In both techniques, the posterior recessus were entered with the shaver and sucked out at the end of the surgery. All patients adhered to a standardized postoperative rehabilitation protocol with partial pain adapted weight bearing for 1–2 weeks and free range of motion.

### Clinical outcome scores and MRI evaluation

Demographic and clinical data including the patient’s age at the time of surgery, the presenting symptoms, range of motion and ligament stability assessed by the ACL ligament score (Lachman test) were collected. In addition, Tegner activity score and Lysholm score were evaluated preoperatively and at final follow-up after a minimum of 12 months.

**Fig. 2:**
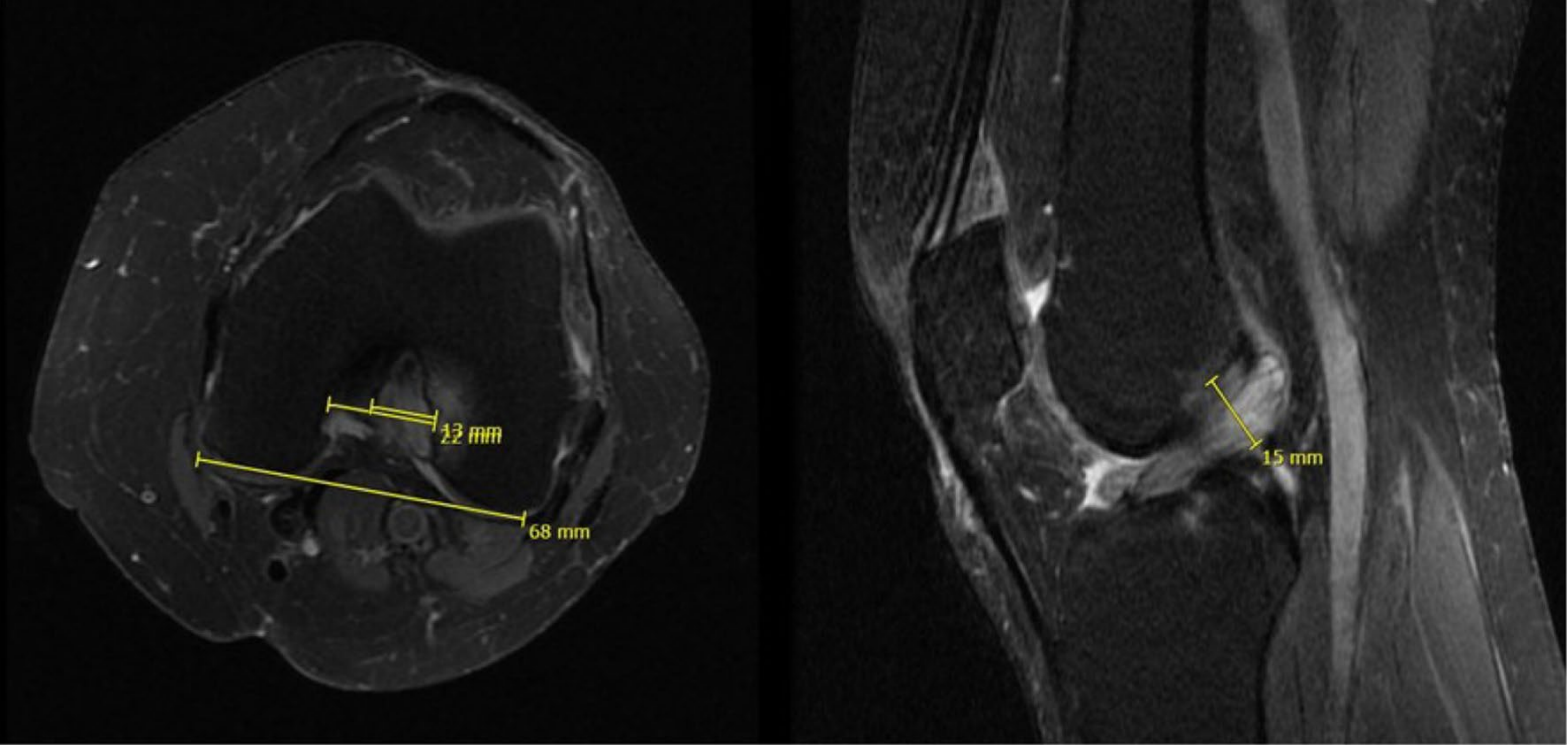
Measurement in the MRI of the MDACL: ratio of the ACL and intercondylar notch width on the axial view and ACL thickness on the sagittal view

MRI evaluation was conducted with the INFINITT Healthcare PACS viewing software (Frankfurt am Main, Germany). Analysis included measurement of the thickness of the ACL (largest diameter on sagittal fat-saturated (fs) proton-density-weighted turbo spin-echo (PDw TSE) sequences), ratio of ACL (largest diameter of the ACL on axial PDw TSE) and intercondylar width, signal intensity of the ACL and presence of bone edema or intraosseous cysts (Fig. 2). MRI analyses were performed before and after surgery at final follow-up. Postoperative MRIs were available from all patients with complete resection of the ACL and from 7 patients who underwent partial resection of the ACL.

### Statistical analysis

Statistical analysis was performed using GraphPad Prism 7 software (GraphPad software, San Diego, USA). Continuous and categorical variables were expressed as mean ± standard deviation (range) and n, respectively. Values of demographics, clinical data/scores and radiological measurements were compared using both paired or unpaired Student’s *t*-test depending on the respective subgroup analysis. *P*-values < 0.05 were considered statistically significant.

## Results

### Demographic data and clinical outcome

Twenty patients (range 22–73 years) with isolated MDACL who were treated between 2018 and 2022 were included in this study. All patients had previously experienced an unsuccessful conservative treatment over 6 months. Ten patients were treated with partial resection (Group I) and 10 patients with complete resection of the ACL (Group II). All patients were diagnosed by MRI and partly by arthroscopic biopsy with specimen taken during the surgical procedure. Among the 20 patients 9 (45%, Group I: *n* = 5 in vs. Group II: *n* = 4) were female and 11 (55%, Group I: *n* = 5 vs. Group II: *n* = 6) were male with a mean age of 49.6 ± 12.2 years (range 22–73; Group I: 51.0 ± 14.1 vs. Group II: 48.1 ± 10.7; ns).

All patients (100%) presented with knee pain, which was most severe at flexion and located posterior. Preoperatively, 19 out of 20 patients had a stable knee with a negative Lachman test (grade I), only 1 patient in Group I showed a slight elongated Lachman test with a firm stop during examination (grade II). At the preoperative examination, range of motion displayed restricted flexion in 15 out of 20 patients with a mean flexion of 119° ± 10° (range, 90–130°; Group I: 119° ± 12° vs. Group II: 120° ± 8°; ns). Extension deficits were not found in the patient cohort.

At final follow-up with a mean of 16.8 ± 8.8 months (range 12–41; Group I: 18.3 ± 9.7 vs. Group II: 15.3 ± 8.0; ns), all patients were pain free.

Postoperatively, positive Lachman tests were noted in all patients (100%) in Group II (*n* = 5 patients with grade II and *n* = 5 patients with grade III). In Group I, 8 patients (80%) showed a negative Lachman test (grade I) and 2 patients (20%) a slightly elongated Lachman test with a firm stop (grade II). Compared to preoperative values, in Group II, IKDC Lachman grades were significantly higher at final follow-up (1.0 ± 0.0 vs. 2.5 ± 0.5; *p* < 0.001). By contrast, patients’ average Lachman grades in Group I showed comparable values prior to and after ACL shrinking procedure (1.1 ± 0.3 vs. 1.2 ± 0.4; ns).

The mean knee flexion at follow-up examination was 132° ± 7° (range, 120 – 140°; Group I: 129° ± 9° vs. Group II: 135° ± 4°; ns). In pairwise comparison, flexion angle increased significantly in both groups (Group I: *p* = 0.0124 and Group II: *p* < 0.001). Both Tegner- and Lysholm Scores improved significantly at follow-up compared to preoperative examination (Tegner: Group I: 3.5 ± 0.5 vs. 4.3 ± 0.8, *p* = 0.011, Group II: 3.5 ± 0.5 vs. 4.6 ± 0.5, *p* = 0.001; Lysholm: Group I: 57.7 ± 7.8 vs. 88.7 ± 12.7, *p* < 0.001; Group II: 55.8 ± 6.1 vs. 93.1 ± 2.7, *p* < 0.001; Table 1).

**Table 1:** Clinical and radiological outcome parameters of all patients with mucoid degeneration of the ACL before and after surgery at follow-up and subdivided into Group I (ACL shrinking) and Group II (complete resection of the ACL)

	All patients			Group I			Group II		
	Baseline	Follow-up	*p* value	Baseline	Follow-up	*p* value	Baseline	Follow-up	*p* value
Flexion (°)	119 ± 10	132 ± 7	< 0.001	119 ± 12	129 ± 9	0.0124	120 ± 8	135 ± 4	< 0.001
Thickness ACL (mm)	15.7 ± 2.4	–	–	14.6 ± 2.4	11.4 ± 3.6	ns	16.7 ± 1.9	–	–
ACL/Inter-condylar ratio	0.61 ± 0.08	–	–	0.65 ± 0.06	0.58 ± 0.22	ns	0.57 ± 0.08	–	–
IKDC Lachman (grades I–IV)	1.1 ± 0.2	1.9 ± 0.8	0.004	1.1 ± 0.3	1.2 ± 0.4	ns	1.0 ± 0.0	2.5 ± 0.5	< 0.001
Tegner	3.5 ± 0.5	4.5 ± 0.7	< 0.001	3.5 ± 0.5	4.3 ± 0.8	0.011	3.5 ± 0.5	4.6 ± 0.5	0.001
Lysholm	56.8 ± 6.9	90.9 ± 9.2	< 0.001	57.7 ± 7.8	88.7 ± 12.7	< 0.001	55.8 ± 6.1	93.1 ± 2.7	< 0.001

Results are shown as mean ± standard deviation

### Radiological outcome

**Fig. 3:**
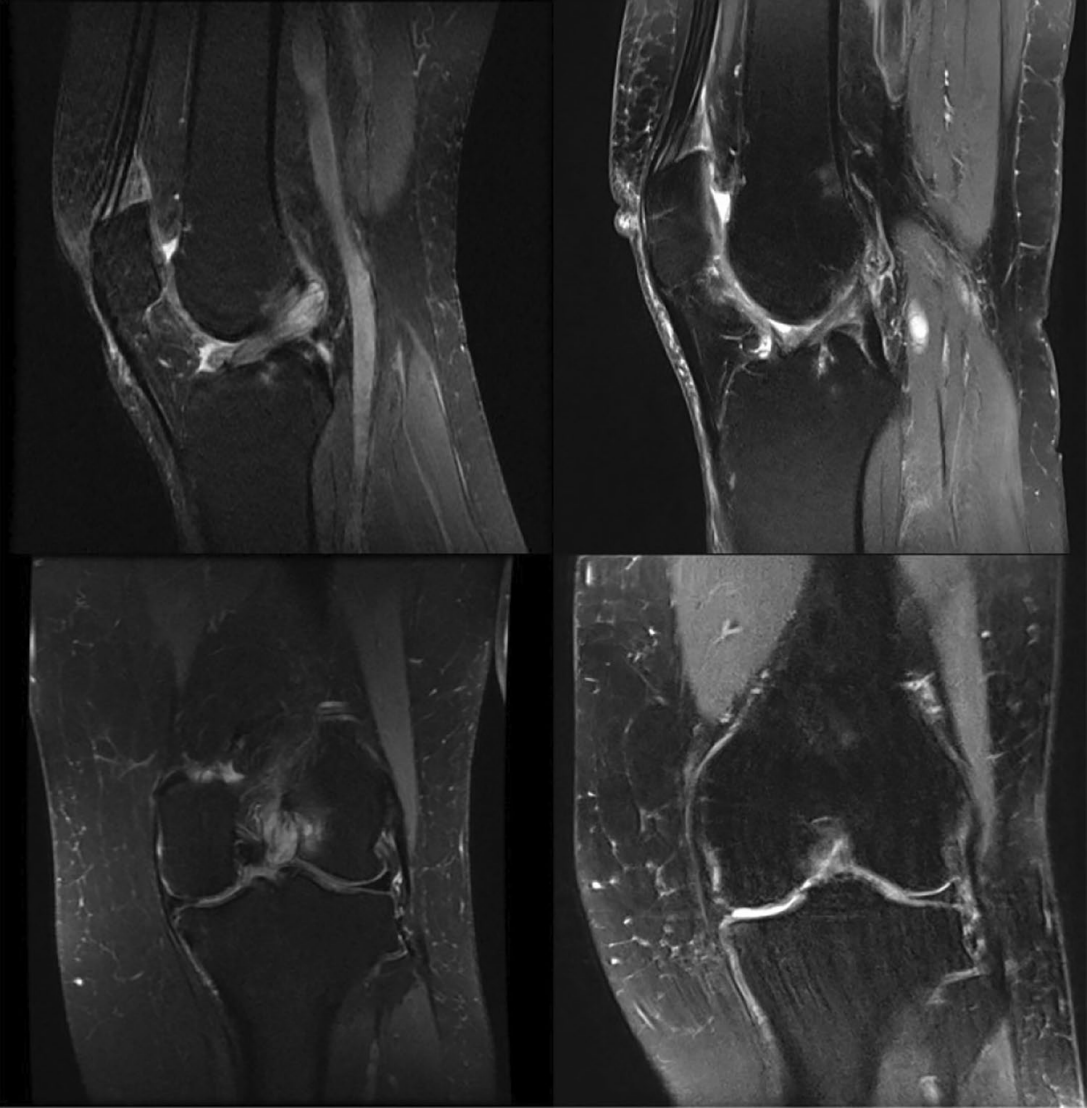
MRI of MDACL before and after treatment of partial resection

Preoperatively, all MRIs showed a hyperintense, bulky ACL, which occupied the intercondylar notch with an increased T2 signal and a mass-like configuration with “celery stalk” sign (Fig. 3). Continuity of the ACL could be observed in all MRI slices prior to surgery. Postoperatively, in Group I, also all patients’ MRIs showed continuity of the ACL (Fig. 3).

On initial MRI scans, mean thickness of ACL was 15.7 ± 2.4 mm (range 11–19 mm) in all patients. On average, ACLs in Group I were measured slightly thinner than in Group II (Group I: 14.6 ± 2.4 mm (range 11–19 mm) vs. Group II: 16.7 ± 1.9 mm (range 14–19 mm), *p* = 0.04).

Postoperatively, Group I showed a mean ACL thickness of 11.4 ± 3.6 mm (range 4–15 mm). Pairwise comparison of thickness values prior to and post-surgery in Group I did not show significant differences.

Mean ACL/intercondylar ratio was 0.61 ± 0.08 (range 0.50–0.71) in all patients. Subgroup analysis revealed a slightly greater ACL/intercondylar ratio in Group I than in Group II in preoperative MRI scans (Group I: 0.65 ± 0.06 (range 0.53–0.71) vs. Group II: 0.57 ± 0.08 (range 0.50–0.71); p = 0.02). After partial resection procedure, mean ACL/intercondylar ratio was 0.58 ± 0.22 (range 0.22–0.93) on follow-up MRI scans in Group I. Again, pairwise comparison of ACL/intercondylar values prior to and post-surgery in Group I did not show significant differences (Table 1).

100% had a distal femur or tibia edema preoperative. 50% of Group I and 60% of Group II showed intraosseous cysts. Postoperative 95% of all patients showed a complete remission of the bone edema and no cysts. Only one patient (Group I) showed a slight and asymptomatic edema in the tibia.

## Discussion

This study demonstrates that both arthroscopic debridement and complete resection of the ACL lead to improvement of clinical and radiological findings in isolated MDACL. However, complete resection of the ACL will result in higher instability of the knee joint and with debridement the ACL can be partly preserved with almost normal ACL function and healthy ligament fibers in the postoperative MRI.

MDACL has been a rare pathological entity because it has been underdiagnosed and confused with other pathologies [7]. It is characterized by infiltration of mucoid-like substance (glycosaminoglycans) interspersed within the substance of ACL causing pain and limited motion of the knee [8]. It seems that not only clinical examination and MRI are necessary for diagnosis, but histology helps in its detection. MDACL was subject of many clinical cases and short heterogeneous series [1–3, 7–18]. All previous studies confirmed that clinical symptoms of MDACL include mostly posterior knee pain with limitation of the range movement and sometimes deteriorated ligament stability[10, 11, 17–19]. Moreover, most patients did not have a trauma prior to the onset of knee pain and their symptoms did not respond to nonsteroidal anti-inflammatory drugs and physiotherapy. This is in line with the present study. All patients developed the symptoms slowly without a distinct injury.

In previous studies arthroscopic total and partial excision of MDACL, combined with or without notchplasty, has been found to result in pain relief and improve in the range of motion of the knee [3, 6, 7]. In our study, we performed 10 partial resections and 10 complete resections and noticed that flexion improved during the postoperative period and secondary MRI findings such as bone edema and cysts were not detectable anymore in the final follow-up. Only one patient showed a residual edema at the tibia. The mean knee motion increased by 13° and all patients regained full flexion after debulking the mucoid ACL. Therefore, both procedures seem to be useful and effective, because in the present study for the first time only patients without concomitant pathologies as meniscal or chondral lesions were included. In contrast, residual pain after partial resection or complete resection of the ACL could not be verified in previous studies and were mostly explained by the presence of concomitant lesions, such as cartilage damage or meniscal tears [6, 20]. However, it is not usual to find patients with isolated MDACL. Kwee et al. demonstrated a strong association between MDACL and cartilage damage, especially in patients > 50 years [21]. Some authors also reported that associated meniscal tears and chondral damage suggest that MDACL may be part of an overall degenerative process [22]. We agree with that opinion because we only found 20 patients with isolated MDACL in 4 years at our specialized knee departments. In these 20 patients all clinical scores improved compared to the preoperative situation. These values are important measures because most of the previous studies have not included clinical scores in the postoperative evaluation. To our knowledge, only three studies have evaluated pre- and post-operative values [2, 14, 20]. However, the issue of instability after ACL resection is a subject to debate; only one study of the published studies about MDACL used a systematic Lachman test or ACL-score postoperatively to assess ACL function after MDACL resection. In that study, 93% of patients had a postoperative anterior laxity and two patients needed a subsequent ACL reconstruction [23]. Unfortunately, the authors did not clarify whether these two patients had developed instability due to chronic stretching after partial resection or due to subtotal or total resection of the mucoid ACL. In the present study, all patients with ACL resection showed an ACL instability in the clinical examination. However, none of them requested an ACL reconstruction because of symptomatic instability in the daily life or while sport activities. After partial resection all patients showed a stable ACL or only a minor instability in the clinical examination and healthy ligament fibers in the follow-up MRI. Therefore, the technique of partial resection seems beneficial compared to the complete resection of the ACL. An additional notchplasty which is considered essential by some authors does not seem to be necessary. In the present study a notchplasty was not performed and excellent results were achieved. This underlines the statement of Motmans and Verheyden who mentioned that notchplasty is not required because thorough debridement of the ACL by itself resolves impingement and thereby the pathology [12]. Ventura et al. did not perform any notchplasty in 25 patients with good results [20]. Lintz et al. performed two notchplasties out of 29 patients but not routinely [3, 23]. Thus, a notchplasty may be only needed in some cases where the notch is stenotic and impinged by osteophytes especially in elderly patients [20]. Therefore, debridement of mucinous substance with partial ACL debulking might be the safest therapeutic option [2, 12]. Moreover young patients are at risk to complain of instability and might need an ACL reconstruction after complete ACL resection [20]. Therefore, if a total removal of the ACL is necessary in a young active patient, an ACL reconstruction should be performed additionally. Some authors recommended this surgery at the same time [3, 17]. We agree with the opinion that ACL reconstruction is necessary in young patients after complete resection of the MDACL but would recommend performing the ACL reconstruction delayed so that the bone can recover, and the edema will disappear.

The present study has several limitations. First, the patient population is small. Second, the follow-up period of only 17 months on average is short, because residual anterior knee instability will most probably lead to a higher rate of subsequent ACL reconstruction over a longer follow-up period, mainly in patients with complete ACL resection. Additionally, a higher rate of ACL ruptures in the patients after partial ACL resection could be possible. Further on a longer follow-up period would give information about a possible rate of recurrence of MDACL after partial resection. Lastly, the inclusion criteria were based on clinical and MRI findings without routine histological verification. However, to our knowledge, this is the first controlled study with patients who have an isolated MDACL to evaluate the effect of partial or complete resection in MDACL specifically.

### Conclusion

Both arthroscopic debridement and complete resection of the ACL lead to improvement of clinical and radiological findings in isolated MDACL. However, complete resection of the ACL will result in higher instability. Therefore, partial resection might be the better treatment option, especially in young patients with MDACL. If a complete resection of the MDACL is necessary in young patients, an ACL reconstruction should be considered.

## Data Availability

Individual patient data will not be available. Individual researchers may contact the corresponding author for access to the original, aggregated and anonymized datasets for research purposes.
